# Heterogeneity in the kinetics of nuclear proteins and trajectories of substructures associated with heterochromatin

**DOI:** 10.1186/1756-8935-4-5

**Published:** 2011-03-18

**Authors:** Lenka Stixová, Eva Bártová, Pavel Matula, Ondřej Daněk, Soňa Legartová, Stanislav Kozubek

**Affiliations:** 1 Institute of Biophysics, Academy of Sciences of the Czech Republic, Brno, Czech Republic; 2 Faculty of Informatics, Masaryk University, Brno, Czech Republic

## Abstract

**Background:**

Protein exchange kinetics correlate with the level of chromatin condensation and, in many cases, with the level of transcription. We used fluorescence recovery after photobleaching (FRAP) to analyse the kinetics of 18 proteins and determine the relationships between nuclear arrangement, protein molecular weight, global transcription level, and recovery kinetics. In particular, we studied heterochromatin-specific heterochromatin protein 1β (HP1β) B lymphoma Mo-MLV insertion region 1 (BMI1), and telomeric-repeat binding factor 1 (TRF1) proteins, and nucleolus-related proteins, upstream binding factor (UBF) and RNA polymerase I large subunit (RPA194). We considered whether the trajectories and kinetics of particular proteins change in response to histone hyperacetylation by histone deacetylase (HDAC) inhibitors or after suppression of transcription by actinomycin D.

**Results:**

We show that protein dynamics are influenced by many factors and events, including nuclear pattern and transcription activity. A slower recovery after photobleaching was found when proteins, such as HP1β, BMI1, TRF1, and others accumulated at specific foci. In identical cells, proteins that were evenly dispersed throughout the nucleoplasm recovered more rapidly. Distinct trajectories for HP1β, BMI1, and TRF1 were observed after hyperacetylation or suppression of transcription. The relationship between protein trajectory and transcription level was confirmed for telomeric protein TRF1, but not for HP1β or BMI1 proteins. Moreover, heterogeneity of foci movement was especially observed when we made distinctions between centrally and peripherally positioned foci.

**Conclusion:**

Based on our results, we propose that protein kinetics are likely influenced by several factors, including chromatin condensation, differentiation, local protein density, protein binding efficiency, and nuclear pattern. These factors and events likely cooperate to dictate the mobility of particular proteins.

## Background

The eukaryotic nucleus is highly compartmentalised. The functional consequences of nuclear compartmentalisation have been described previously in both fixed and live cells [1-3]. Generally, chromatin consists of histones wrapped with DNA and the many proteins that are directly responsible for proper nuclear functions, such as replication, transcription, splicing, and DNA repair. Nuclear processes proceed in specific compartments, such as nuclear foci, transcription and replication factories, or nuclear speckles (summarised in [4,5]). Moreover, interphase chromosomes are arranged into chromosome territories, which can intermingle to some degree [4,6,7]. Centromeres and telomeres are also essential structures for chromosome function. Centromeres are the sites of mitotic spindle attachment and are required for cell division [8]. Telomeres, which include the shelterin proteins (telomeric-repeat binding factor 1 (TRF1), TRF2, protection of telomeres 1 (POT1), tripeptidyl peptidase 1 (TPP1), Ras-related protein 1 (RAP1), and TRF1-interacting nuclear factor 2 (TIN2)), protect the ends of chromosomes and are essential for chromosome stability [9,10]. Another prominent nuclear structure is the nucleolus, which is the largest transcription factory and is necessary for the synthesis of ribosomal subunits [11-13]. Like the rest of the genome, nucleoli are remarkably compartmentalised. The nucleolar region consists of the fibrillar centre (FC), the dense fibrillar component (DFC), and the granular component (GC). The boundaries of nucleoli are surrounded by clusters of centromeric heterochromatin, called chromocentres [14]. The nucleolar organiser regions (NORs) of specific acrocentric chromosomes are responsible for the structural and functional properties of nucleoli [15,16]. Many specific proteins preferentially localise to the boundary between the FC and DFC, which is thought to be the site of transcription of ribosomal genes. The transcription machinery used for rRNA synthesis includes RNA polymerase I (RNA pol I) and upstream binding factor (UBF) [17,18]. Gorski *et al. *[19] measured the dynamic nature of these important nucleolar proteins using fluorescence recovery after photobleaching (FRAP). In that study, the fluorescence recovery kinetics of several RNA pol I subunits in the G1 and S phases of the cell cycle displayed biphasic behaviour, characterised by fast fluorescence increase followed by a gentle fluorescence recovery phase [19]. These authors also showed that the RNA polymerase I recovery kinetics decrease as cell cycle-dependent transcription increases. The transcription of RNA polymerase I can be suppressed by exposure to a variety of stimuli. Actinomycin D is a widely used drug that intercalates into double stranded DNA and blocks the transcription elongation activity of all three polymerases (I, II, III) [20,21]. Therefore, actinomycin D is a useful agent that enables the study of gene silencing and corresponding epigenetic events.

Green fluorescent protein (GFP) technologies combined with microscopy have opened a new avenue for studying the mobility of tagged proteins in living cells. However, most methods are limited by the confocal optical resolution of about 200 nm laterally and 600 nm axially [22]. Thus, several microscopy techniques have been developed to improve the microscope resolution to 10 to 20 nm. The new methods include 4Pi microscopy [23], localisation microscopy approaches [22], three-dimensional structured illumination microscopy (3D-SIM) [24], and stimulated emission depletion microscopy (STED) [25]. Photobleaching methods are used to analyse the kinetic properties of particular proteins [1,26]. Many chromatin-related proteins have been studied using the FRAP technique to measure local protein dynamics, which are important functional characteristics of these proteins. For example, two distinct kinetic pools for Polycomb group-related protein, B lymphoma Mo-MLV insertion region 1 (BMI1), have been described [27]. Similarly, Wang *et al. *[28] showed the energy-dependent heterogeneous movement of telomeres. In other studies, it has been reported that heterochromatin protein 1 (HP1α, HP1β, HP1γ) accumulates more rapidly into euchromatin-rich nuclear regions than the HP1 subtypes of highly condensed heterochromatin foci [29,30]. The recovery of HP1α protein is faster in pluripotent mouse embryonic stem cells (mESCs) than in differentiated cells [31]. Furthermore, the mobile fraction of histones H2B and H3 is low compared to HP1 subtypes. However, an increased recovery after photobleaching of H2B-GFP and H3-yellow fluorescent protein (YFP) was measured in mESCs relative to differentiated cells. This implies that the kinetics of chromatin-related proteins are cell-type specific and dependent on the level of chromatin condensation [31].

Previous studies have provided interesting data for functionally important features of chromatin-related proteins. The observations described above have led to additional questions. Is the association of particular proteins with nuclear domains of specific function the most important criterion responsible for their kinetic properties or trajectories? Could molecular weight or protein binding efficiency also influence protein diffusion into a photobleached region? We analysed the trajectories and kinetics of several chromatin-related proteins and determined the relationships between protein nuclear arrangement, molecular weight, and recovery kinetics. In addition, we asked whether the trajectories and kinetics of particular proteins change in response to histone hyperacetylation by the histone deacetylase (HDAC) inhibitor trichostatin A (TSA) or after suppression of transcription by actinomycin D.

## Results

### Cellular patterns of proteins studied

**Figure 1:**
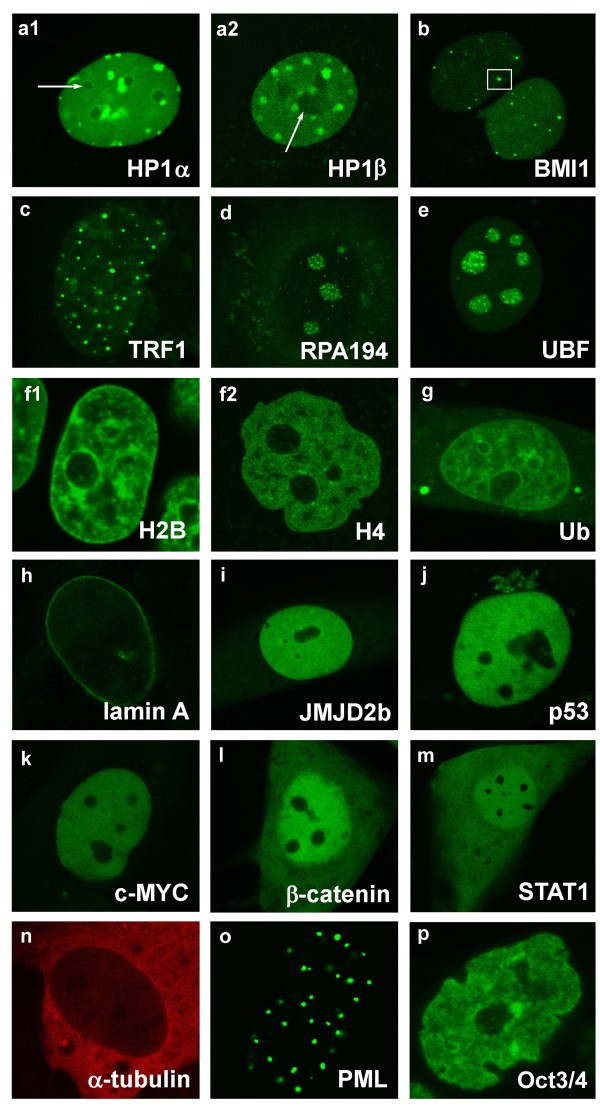
**Cellular patterns of selected proteins**. The dynamics of selected proteins were studied using GFP technology combined with fluorescence recovery after photobleaching (FRAP). The following proteins were analysed: heterochromatin protein 1 (HP1α) (a1), (HP1β) (a2), B lymphoma Mo-MLV insertion region 1 (BMI1) (b), telomeric-repeat binding factor 1 (TRF1) (c), RNA polymerase I large subunit (RPA194) (d), upstream binding factor (UBF) (e), histones H2B (f1), H4 (f2), ubiquitin (Ub) (g), A-type lamins (h), histone demethylase JMJD2b (i), tumour suppressor p53 (j), oncoprotein c-MYC (k), β-catenin (l), STAT1 (m), α-tubulin (n), PML protein (o), and Oct3/4 (p).

In this study, we analysed the dynamics of a select group of proteins, which displayed the following cellular patterns: subtypes of heterochromatin protein 1 (HP1α and HP1β) preferentially accumulated at foci that colocalised with centromeric clusters, called chromocentres (foci). HP1α (Figure 1 a1), and HP1β (Figure 1 a2), were observed away from chromocentres (euchromatin) and within nucleoli (arrows) (Figure 1a). The BMI1 protein is a member of the protein regulator of cytokinesis 1 (PRC1) protein complex, which accumulated at Polycomb bodies (PcG) (frame in Figure 1b), but were also observed outside of PcG bodies (Figure 1b). The TRF1 protein is a member of the shelterin complex associated with telomeres and was preferentially bound to clusters of telomeres (Figure 1c). RNA polymerase I large subunit (RPA194), occupied a compartment of nucleoli (Figure 1d). UBF is a member of the high mobility group (HMG) proteins and exists as two spliced variants (UBF1 and UBF2), which act as transcription enhancers. Thus, UBF was found at the active promoters of ribosomal genes (rDNA) [11] (Figure 1e). We also analysed the fluorescence recovery after photobleaching of histones H2B (Figure 1 f1) and H4 (Figure 1 f2), representing the basic core histones responsible for general higher-order chromatin structure (summarised in [4]). By analysing the H2B pattern, it was possible to identify chromatin condensation (heterochromatin) at the nuclear periphery and around nucleoli. Moreover, it was possible to distinguish potential dark regions of euchromatin (Figure 1 f1). The expression of ubiquitin-GFP (Ub) was used to visualise highly ubiquitinated regions of the genome, but this signal also appeared in the cytoplasm (Figure 1g). A-type lamins (lamin A and lamin C) were preferentially located at the nuclear periphery and partially in the nuclear interior (green signal inside nucleus) (Figure 1h). We also analysed the dynamics of full-length histone demethylase, JMJD2b, which was equally distributed within interphase nuclei (Figure 1i). Similarly, the tumour suppressor p53 (Figure 1j), oncoprotein c-MYC (Figure 1k), β-catenin (Figure 1l), and ESC pluripotency-related signal transducer and activators of transcription 1 (STAT1) protein (Figure 1m) were homogeneously distributed in the nucleoplasm. Similar to α-tubulin (Figure 1n), STAT1 and β-catenin appeared in the cytoplasm. Promyelocytic leukaemia (PML) protein accumulated at PML bodies (Figure 1o) and the octamer-binding transcription factor (Oct)3/4 protein (Figure 1p) was homogeneously dispersed throughout the nucleoplasm in pluripotent mESCs. Taking into account the dynamic properties of all the proteins studied and their molecular weights, we asked whether the nuclear patterns influenced the kinetic properties of these proteins, or if molecular weight was the sole factor.

### Correlation between molecular weight and kinetics of chromatin-related proteins

**Figure 2:**
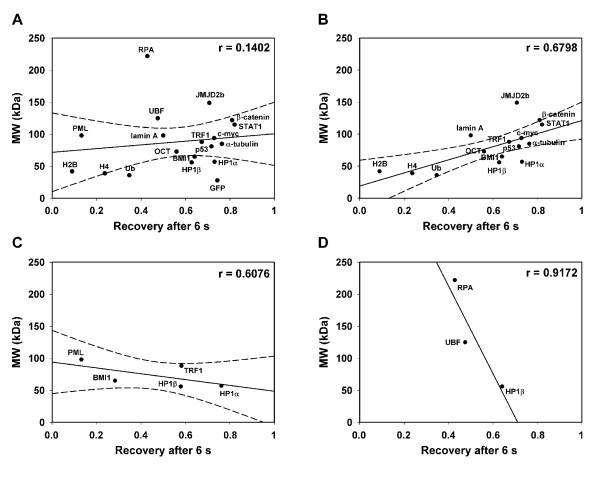
**Test of correlations between molecular weight and recovery time after photobleaching**. (a) The kinetic properties after photobleaching were studied for the following proteins: heterochromatin protein 1α (HP1α), HP1β, B lymphoma Mo-MLV insertion region 1 (BMI1), telomeric-repeat binding factor 1 (TRF1), RNA polymerase I large subunit (RPA194), upstream binding factor (UBF), H2B, H4, ubiquitin (Ub), lamin A (central), JMJD2b, p53, c-MYC, β-catenin, STAT1, α-tubulin, PML, and Oct3/4. α-Tubulin was tagged with mCherry (35 kDa) and other proteins were tagged with GFP (28 kDa). Molecular weights of individual proteins were compared by correlation analysis with the level of relative fluorescence 6 s after photobleaching [*R*_6_]. (b) A correlation between molecular weight and fluorescence recovery of proteins was found for proteins that were evenly dispersed throughout the nucleoplasm: HP1α, HP1β, BMI1, TRF1, H2B, H4, Ub, lamin A (central), JMJD2B, p53, c-MYC, β-catenin, STAT1, α-tubulin, Oct3/4. (c) No correlation between molecular weight and fluorescence recovery for proteins that were accumulated into foci: HP1α, HP1β, BMI1, TRF1, and PML. (d) No correlation was detected between molecular weight and fluorescence recovery for proteins accumulated into nucleoli: HP1β, RPA194, UBF. Pearson's correlation coefficient for (n-2) = 17 is 0.456; for (n-2) = 13 is 0.514; for (n-2) = 3 is 0.878 and for (n-2) = 1 is 0.997. These values are for α = 0.05. Regression lines surrounded by 95% confidence intervals (dashed curves) are shown in all panels except panel (d); to unify the axis scale, confidence intervals are not shown.

We used regression analysis to determine whether the kinetic properties of chromatin-related protein are influenced by nuclear pattern and molecular weight. We compared the molecular weights of 18 individual proteins (tagged by GFP or mCherry; see Methods) with their recovery of fluorescence 6 s after photobleaching. Additionally, we analysed the recovery of GFP; after 6 s [*R*_6_] it was 74% in the nucleus and 81% in the cytoplasm (data not shown). We found no correlation when we studied all proteins irrespective of their nuclear patterns (Figure 2a). However, when we grouped proteins according to nuclear pattern, distinctions were observed between proteins that were dispersed throughout the nucleoplasm and those that accumulated at foci or nucleoli. For evenly distributed proteins, the nuclear pattern acted together with molecular weight to influence recovery time after photobleaching (Figure 2b). We next determined whether eliminating histones from this analysis influenced this correlation. The following values were obtained from regression analysis: analysis involving histones had a Pearson's correlation coefficient *r *= 0.68 and analysis without histones showed *r *= 0.57 (data not shown). Both values are significant for α = 0.05; thus, histone elimination did not influence the results.

In contrast, we showed that there was no significant correlation between molecular weight and fluorescence recovery for proteins that accumulated at foci (Figure 2c) or nucleoli (Figure 2d). These results indicate that molecular weight is not the sole factor in determining protein kinetics, but that other factor can influence the dynamics of some biologically active molecules.

### Trajectories of HP1β, BMI1, and TRF1 foci are influenced by histone hyperacetylation and suppression of transcription

**Figure 3:**
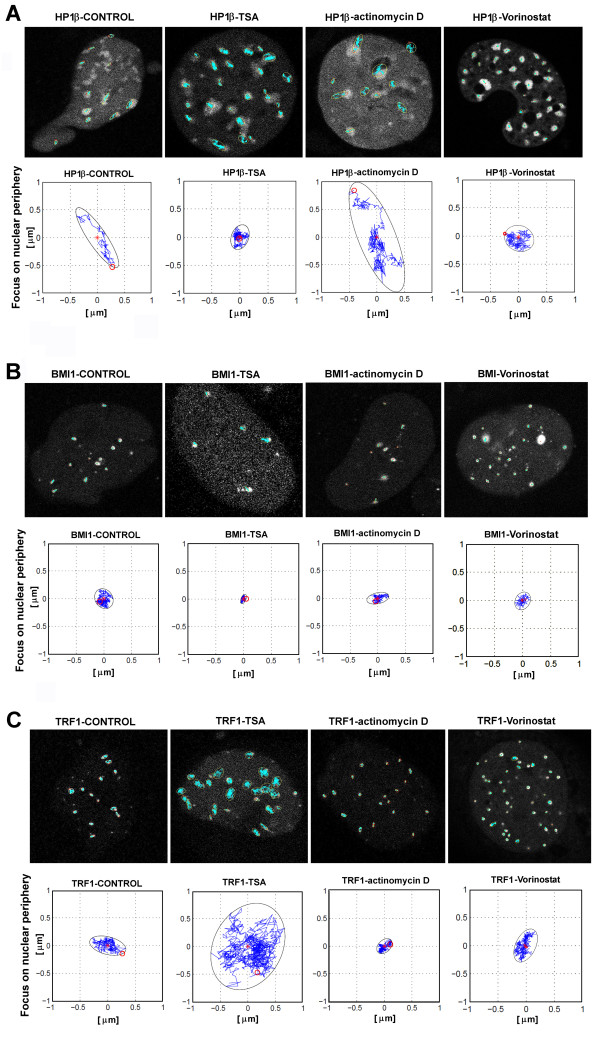
**Heterogeneity in the trajectory of heterochromatin protein 1β (HP1β), B lymphoma Mo-MLV insertion region 1 (BMI1), and telomeric-repeat binding factor 1 (TRF1) foci at the nuclear periphery**. Real-time monitoring of the foci trajectories is shown for HP1β (a), BMI1 (b), and TRF1 (c) foci. Trajectories were mapped for foci that occupy the nuclear periphery. Heterogeneity in movement of all foci (blue tracks) within an individual cell nucleus (grey) and events is shown. HP1β, BMI1, and TRF1 kinetics was studied in control cells, after TSA, actinomycin D, and vorinostat treatment. Observation was performed over 20 min, scanning was performed every 2 s. Each trajectory (blue curves) is bound by an ellipse enclosing the area of movement; the red star is the centre of the ellipse and the red circle is the start point of movement.

**Figure 4:**
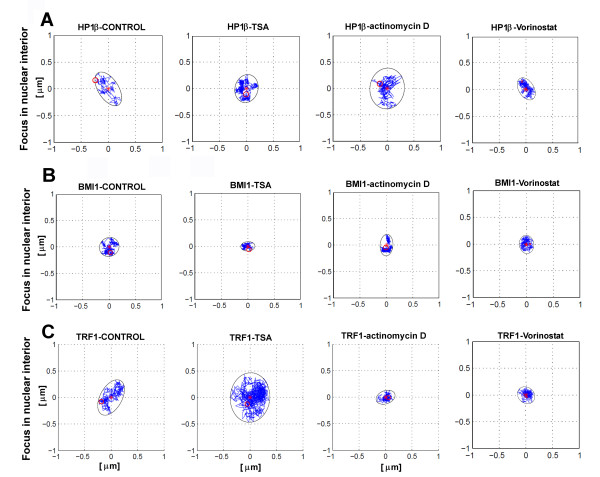
**Heterogeneity in the trajectory of heterochromatin protein 1β (HP1β), B lymphoma Mo-MLV insertion region 1 (BMI1), and telomeric-repeat binding factor 1 (TRF1) foci at the nuclear interior**. Real-time monitoring of the foci trajectories is shown for HP1β (a), BMI1 (b), and TRF1 (c) foci. Trajectories were mapped for foci that occupy the nuclear interior. HP1β, BMI1, and TRF1 kinetics was studied in control cells, after TSA, actinomycin D, and vorinostat treatment. Observation was performed over 20 min, scanning was performed every 2 s. Each trajectory (blue curves) is bound by an ellipse enclosing the area of movement; the red star is the centre of the ellipse and the red circle is the start point of movement.

**Figure 5:**
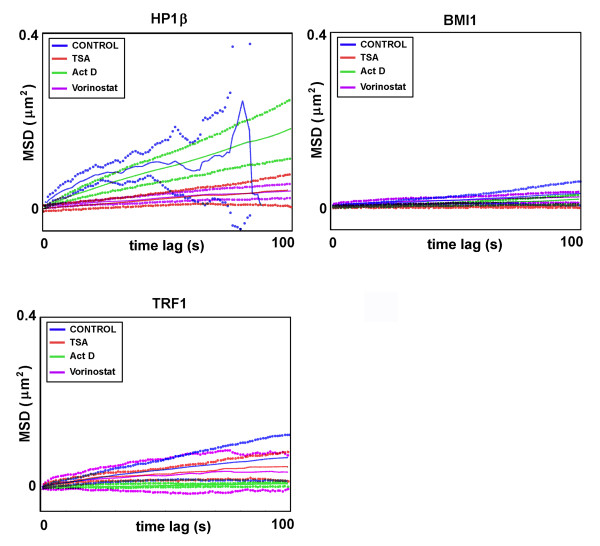
**Mean square displacement (MSD) plots describe the foci movement**. The simulated MSD is represented by solid line, the theoretical MSD is not shown, but the MSD ± SD are dashed lines.

Real-time optical monitoring of cellular trajectories showed differences in the movements of HP1β, BMI1, and TRF1 foci when cells were treated with TSA, vorinostat, or actinomycin D (Figures 3, 4, 5). Mouse fibroblasts are relatively flat; thus, we analysed the trajectories in two dimensions. We studied the trajectories of whole foci within the cell nucleus, but not the trajectories of selected protein points within individual foci. Heterogeneity was observed in the trajectories of HP1β, BMI1, and TRF1 in all cases tested, but to a lesser extent for BMI1 in control cells (Figures 3, 4, 5). We also observed differences in the trajectories, especially when we distinguished between foci positioned in the nuclear interior or the nuclear periphery (Figures 3 and 4). When we studied the tracks of HP1β at the nuclear periphery, especially after actinomycin D treatment, the area of the minimal enclosing ellipse of individual HP1β foci was increased (Figure 3a and Table 1). The trajectories of BMI1 foci at the nuclear periphery were slightly reduced by TSA treatment (Figure 3b and Table 1). However, the large-scale area of minimal enclosing ellipses for TRF1 foci at the nuclear periphery were increased after TSA addition, while the TRF1 trajectories were reduced when cells were treated with actinomycin D (Figure 3c and Table 1). Changes in the TRF1 trajectories correspond well to changes in global transcription and chromatin condensation, which are increased after TSA, but decreased after actinomycin D treatment.

**Table 1:** Area of heterochromatin protein 1β (HP1β), B lymphoma Mo-MLV insertion region 1 (BMI1), and telomeric-repeat binding factor 1 (TRF1) foci

	**Area of minimal enclosing ellipse (μm**^**2**^**)**		
	
	HP1β	BMI1	TRF1
Peripheral foci: control	0.22 ± 0.12 (n = 10)	0.17 ± 0.09 (n = 4)	0.19 ± 0.06 (n = 8)
Peripheral foci: TSA	0.13 ± 0.10 (n = 19)	0.02 ± 0.02 (n = 12)	0.7 ± 0.54 (n = 15)
Peripheral foci: actinomycin D	0.59 ± 0.48 (n = 4)	0.09 ± 0.05 (n = 5)	0.08 ± 0.05 (n = 20)
Peripheral foci: vorinostat	0.17 ± 0.10 (n = 23)	0.17 ± 0.22 (n = 13)	0.28 ± 0.47 (n = 29)

Central foci: control	0.15 ± 0.07 (n = 7)	0.11 ± 0.05 (n = 10)	0.27 ± 0.21 (n = 8)
Central foci: TSA	0.16 ± 0.09 (n = 16)	NA (n = 0)	0.65 ± 0.42 (n = 8)
Central foci: actinomycin D	0.69 ± 0.46 (n = 6)	0.07 ± 0.06 (n = 5)	0.05 ± 0.02 (n = 11)
Central foci: vorinostat	0.21 ± 0.12 (n = 13)	0.09 ± 0.03 (n = 10)	0.15 ± 0.17 (n = 18)

Area mean: control	0.19 ± 0.11	0.13 ± 0.07	0.23 ± 0.16
Area mean: TSA	0.14 ± 0.09	-	0.68 ± 0.49
Area mean: actinomycin D	0.65 ± 0.44	0.08 ± 0.05	0.06 ± 0.04
Area mean: vorinostat	0.18 ± 0.11	0.14 ± 0.17	0.23 ± 0.39

Mean values ± standard deviations were calculated for all foci found in one living cell. Number of foci analysed is shown in brackets. Central foci were positioned in <60% of nuclear radius, as peripheral foci were considered those located at >60% the nuclear radius. TSA = trichostatin A.

In the nuclear interior, the trajectory of HP1β was not remarkably influenced by TSA, compared to the control. However, actinomycin D treatment subtly increased the area occupied by HP1β (Figure 4a and Table 1). On average, the trajectory of BMI1 at the nuclear interior was relatively stable after TSA and actinomycin D treatment (Figure 4b and Table 1). In addition, we observed significant changes in interior TRF1 foci after TSA and actinomycin D treatment (Figure 4c and Table 1): TSA prolonged the track of TRF1, while actinomycin D reduced the movement of TRF1 that appeared in the nuclear interior (Figure 4c and Table 1). The changes in the average area occupied by the foci studied were accompanied by changes in average velocities (compare Table 1 with Table 2). However, different trends were observed; increased average velocity was not always accompanied by a larger area of minimal enclosing ellipse, and *vice versa*.

**Table 2:** Average velocity of heterochromatin protein 1β (HP1β), B lymphoma Mo-MLV insertion region 1 (BMI1), and telomeric-repeat binding factor 1 (TRF1) foci

	Average velocity (μm/s)		
	
	HP1β	BMI1	TRF1
Peripheral foci: control	0.034 ± 0.007	0.017 ± 0.003	0.024 ± 0.005
Peripheral foci: TSA	0.024 ± 0.011	0.012 ± 0.005	0.046 ± 0.012
Peripheral foci: actinomycin D	0.024 ± 0.003	0.016 ± 0.005	0.021 ± 0.015
Peripheral foci: vorinostat	0.020 ± 0.003	0.020 ± 0.007	0.026 ± 0.010

Central foci: control	0.036 ± 0.018	0.015 ± 0.003	0.021 ± 0.004
Central foci: TSA	0.028 ± 0.017	NA	0.050 ± 0.020
Central foci: actinomycin D	0.022 ± 0.003	0.013 ± 0.003	0.018 ± 0.003
Central foci: vorinostat	0.020 ± 0.004	0.017 ± 0.003	0.026 ± 0.010

Velocity mean: control	0.035 ± 0.012	0.016 ± 0.003	0.023 ± 0.005
Velocity mean: TSA	0.026 ± 0.014	-	0.047 ± 0.015
Velocity mean: actinomycin D	0.023 ± 0.003	0.015 ± 0.005	0.020 ± 0.010
Velocity mean: vorinostat	0.020 ± 0.004	0.018 ± 0.005	0.026 ± 0.010

NA indicates that it was not possible to recognise centrally positioned foci using automated computer analysis.

TSA = trichostatin A.

Vorinostat, or suberoylanilide hydroxamic acid (SAHA), is a potential clinical inhibitor of HDACs. Thus, in parallel with TSA, we analysed the effect of vorinostat on the GFP-HP1β, GFP-BMI1, and GFP-TRF1 trajectories (Figures 3 and 4). Generally, we observed that vorinostat had a more moderate effect on protein trajectories than TSA (Figures 3 and 4). In the case of HP1β vorinostat treatment reduced the area of minimal enclosing ellipse at both the nuclear periphery and interior (Figures 3 and 4). Vorinostat was also found to effect protein movement when we analysed the diffusion coefficient (*D*) and mean square displacement (MSD) (Table 3 and Figure 5). Taken together, these data show that vorinostat decreased the value of *D *in all three proteins analysed (Table 3). These trends are also expressed by MSD (see Figure 5).

**Table 3:** Diffusion coefficient of heterochromatin protein 1β (HP1β), B lymphoma Mo-MLV insertion region 1 (BMI1), and telomeric-repeat binding factor 1 (TRF1) foci

Foci	**Diffusion coefficient *D *(10**^**-4 **^**μm**^**2**^**/s)**		
	
	HP1β	BMI1	TRF1
Control	3.05	0.74	1.61
TSA	1.05	0.04	0.95
actinomycin D	4.28	0.42	0.11
vorinostat	0.90	0.37	0.73

TSA = trichostatin A.

The diffusion coefficient *D *for HP1β differed between control cells and those treated by HDAC inhibitors (TSA and vorinostat) and actinomycin D (Table 3). Conversely, changes in BMI1 diffusion were not so pronounced after selected treatments, whereas the diffusion coefficient for TRF1 significantly changed after all treatments (Table 3). Conclusions related to diffusion coefficient are closely related to trends expressed by the MSD (Figure 5).

### Recovery of fluorescence after photobleaching of HP1β, BMI1, and TRF1 proteins after HDAC inhibition and suppression of transcription by actinomycin D

**Figure 6:**
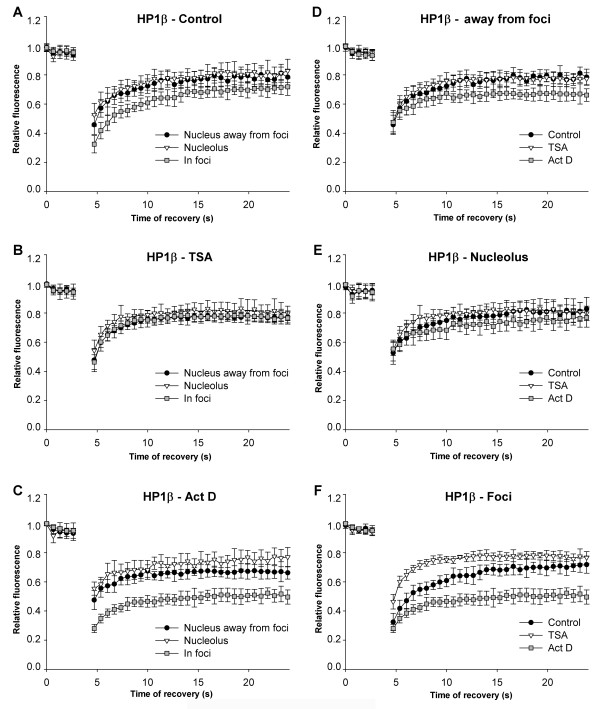
**Heterochromatin protein 1β (HP1β) dynamics in different nuclear regions**. The kinetic properties of HP1β proteins are provided for HP1β accumulated in foci of heterochromatin, nuclear regions outside of foci (likely euchromatin), and HP1β accumulated in nucleoli. This analysis was performed in (a) control MEFs, (b) TSA-treated MEFs, and (c) actinomycin D-treated MEFs. Comparison of HP1β in control (black dots), TSA-treated cells (triangles), and actinomycin D-stimulated (squares) cells. Proteins were analysed (d) in euchromatin or away from foci; e.g. in interchromatin space; (e) nucleoli, and (f) in foci of heterochromatin.

**Figure 7:**
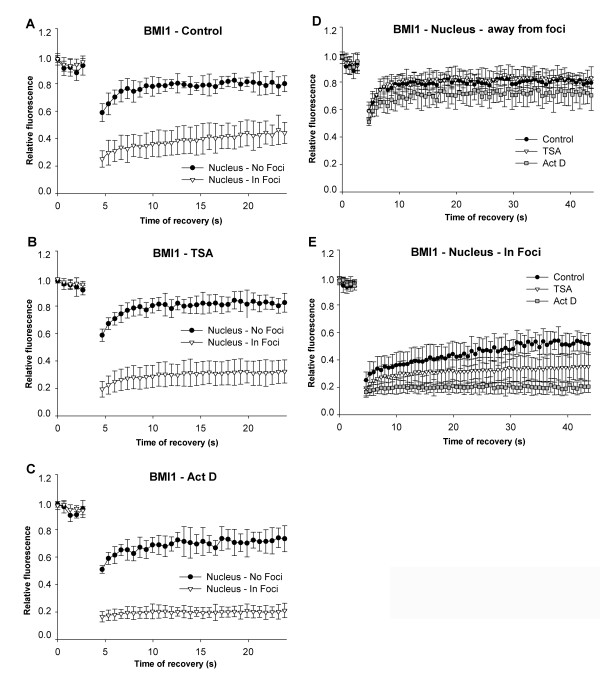
**B lymphoma Mo-MLV insertion region 1 (BMI1) dynamics in different nuclear regions**. Fluorescence recovery time after photobleaching was studied for BMI protein accumulated in PcG bodies and outside of PcG bodies in (a) control MEFs; (b) TSA-treated MEFs; and after actinomycin D treatment (c). Comparison of BMI1 in control (black dots), TSA-treated (triangles), and actinomycin D-stimulated (squares) cells in nuclear regions (d) devoid of PcG bodies, and (e) in PcG bodies.

**Figure 8:**
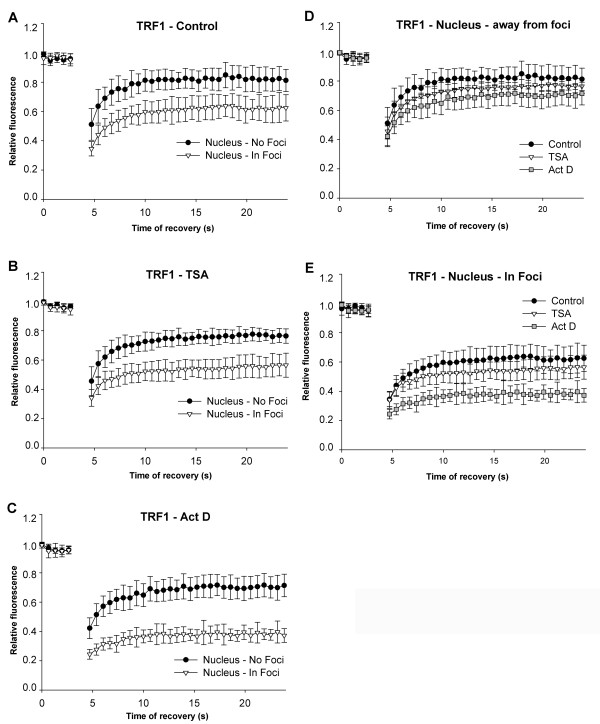
**Telomeric-repeat binding factor 1 (TRF1) dynamics at telomeric regions and nuclear regions away from telomeres**. Fluorescence recovery time after photobleaching was studied for TRF1 protein accumulated in telomeric clusters (triangle) and away from telomeres (black dots). The analysis was performed in (a) control MEFs; (b) TSA-treated MEFs and (c) after actinomycin D treatment. Additional comparison was performed for TRF1 in control (black dots), TSA-treated (triangles), and actinomycin D-stimulated (squares) cells in nuclear regions (d) absent of telomeric clusters (unbounded protein), and (e) for TRF1 foci associated with clusters of telomeres.

We next asked whether histone hyperacetylation, caused by the HDAC inhibitor TSA, or inhibition of transcription by actinomycin D changed the kinetics of heterochromatin-related proteins, such as HP1β (Figure 6), BMI1 (Figure 7), and TRF1 (Figure 8). Immortalised mouse embryonic fibroblasts (MEFs) were transfected with plasmids encoding HP1β or TRF1. Human U2OS cells stably expressing BMI1 were also used in this analysis. HP1β preferentially accumulated in centromeric clusters [32]. As it is a Polycomb-related protein (PcG), BMI1 appeared in Polycomb bodies [33], while TRF1, as a member of the shelterin protein complex, accumulated at telomeric clusters (summarised in [34]). The recovery times of the selected proteins after photobleaching were measured in approximately 20 identical cells, either throughout the genome or when the proteins were accumulated in foci or bodies. Moreover, since HP1β has been detected in nucleoli [35], we also analysed the fluorescence recovery after photobleaching of nucleolar HP1β. Generally, proteins equally dispersed throughout the nucleoplasm recovered more rapidly than proteins that were accumulated at foci (Figures 6, 7, 8). When we bleached half of a nucleolus, we found that movement was the same regardless of whether the nucleolus had a high or low number of foci (data not shown).

We confirmed that the fluorescence recovery kinetics of HP1β is more rapid in euchromatin than in heterochromatic chromocentres ([30] and Figure 6a). However, the recovery time after photobleaching was similar for HP1β in euchromatin away from foci (recovery after 10 s [*R*_10_] was 72%) and in nucleoli [*R*_10_] = 74% (Figure 6a). Interestingly, after TSA treatment, the curves were identical for HP1β in euchromatin ([*R*_10_] = 76%), in heterochromatic foci ([*R*_10_] = 75%), and in the nucleolus ([*R*_10_] = 79%) (Figure 6b). When transcription was suppressed by actinomycin D, the fluorescence recovery kinetics of HP1β was significantly slower only in chromocentres ([*R*_10_] = 47%) (foci) (Figure 6c). We did not observe statistically significant changes when we compared the kinetics of HP1β in euchromatin (Figure 6d) and within nucleoli (Figure 6e) after TSA and actinomycin D treatment. However, the recovery time after photobleaching for the HP1β in foci significantly decreased after actinomycin D and increased after TSA addition (Figure 6f). Thus, we confirmed the effect of TSA on heterochromatin regions, which has also been published by Cheutin *et al. *[30]).

Fluorescence recovery of BMI1 was rapid when it was diffuse in the nucleoplasm ([*R*_10_] = 78%) (Figure 7a), and slower when it was accumulated in PcG bodies (foci) ([*R*_10_] = 36%) (Figure 7a). After TSA treatment, this trend was similar, but the differences between the BMI1 molecules outside ([*R*_10_] = 80%) and within PcG foci ([*R*_10_] = 28%) were more pronounced (Figure 7b). Moreover, actinomycin D significantly reduced the fluorescence recovery time after photobleaching of BMI1 protein accumulated in foci ([*R*_10_] = 20%) (Figure 7c). We measured a slower recovery for BMI1 away from PcG bodies when the cells were treated with actinomycin D (Figure 7d), but this was not statistically significant. Interestingly, the BMI1 in PcG bodies recovered more slowly after TSA treatment, but the difference was not as strong as after actinomycin D treatment (Figure 7e).

The telomeric TRF1 protein also showed significant differences depending on whether or not TRF1 was accumulated in foci. TRF1 foci are likely clusters consisting of several telomeres, consistent with the observation that cells expressing GFP-TRF1 had fewer TRF1-positive foci than telomeres. We measured [*R*_10_] = 81% for TRF1 outside of foci (likely the unbound form), and [*R*_10_] = 60% for TRF1 localised to telomere clusters (Figure 8a). Changes in TRF1 fluorescence recovery time were also observed in cells treated with TSA or actinomycin D (Figure 8b,c). After TSA treatment, [*R*_10_] *= *72% for unbound TRF1 and [*R*_10_] *= *52% for TRF1 within foci (Figure 8b). Similarly, when cells were treated with actinomycin D, [*R*_10_] = 64% for TRF1 outside of foci, but for TRF1 within foci, [*R*_10_] = 37% (Figure 8c). We also compared TRF1 fluorescence recovery after photobleaching in untreated control cells, and TSA-treated and actinomycin D-treated cells. After actinomycin D treatment, fluorescence recovery time after photobleaching was not significantly reduced for TRF1 outside of foci (Figure 8d). However, TRF1 recovery was significantly reduced within foci, particularly in cells treated with actinomycin D (Figure 8e).

**Figure 9:**
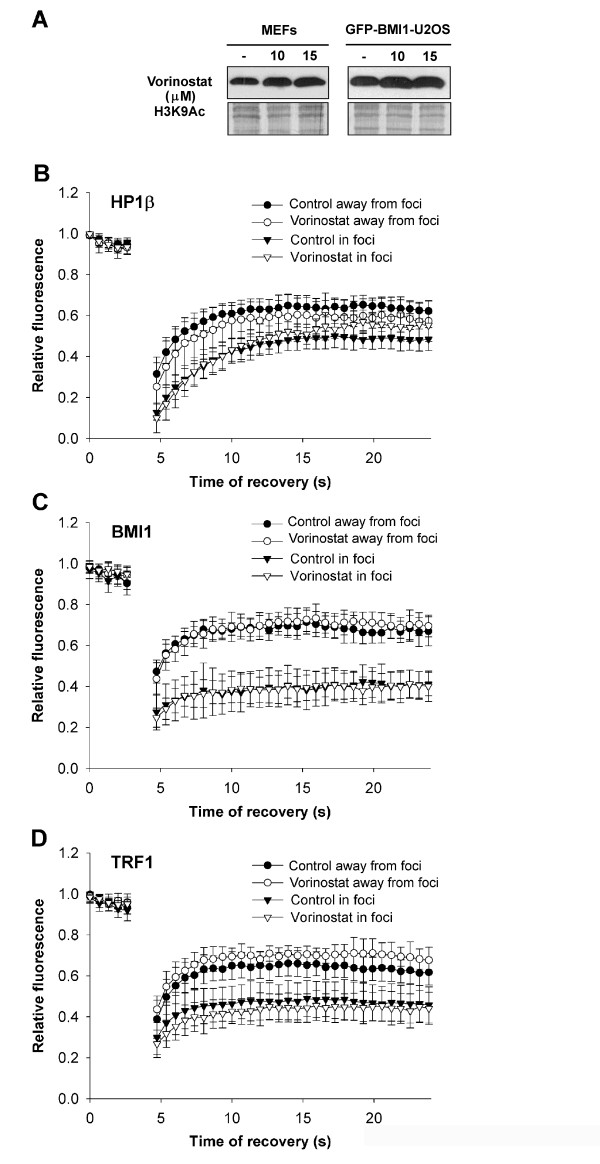
**Heterochromatin protein 1β (HP1β), B lymphoma Mo-MLV insertion region 1 (BMI1), and telomeric-repeat binding factor 1 (TRF1) fluorescence recovery after vorinostat treatment**. (a) Western blot analysis of H3K9 acetylation in MEFs and GFP-BMI-U2OS cells treated by vorinostat. (b) Fluorescence recovery of HP1β accumulated into foci or outside of foci in control and vorinostat-treated MEFs. (c) Fluorescence recovery after photobleaching (FRAP) data of BMI1 accumulated into PcG bodies or outside of PcG bodies in control and vorinostat-treated GFP-BMI-U2OS cells. (d) Fluorescence recovery of TRF1 associated with telomeres or dispersed in interchromatin space of control and vorinostat-treated MEFs.

We also measured the effect of vorinostat on the fluorescence recovery of selected proteins (Figure 9). The inhibitory effect of vorinostat was confirmed by western blot showing increased H3K9 acetylation (Figure 9a). Despite the effect of vorinostat on histone acetylation, FRAP analysis showed only subtle changes in the fluorescence recovery of HP1β, BMI1, and TRF1 when we distinguished proteins in foci and away from foci (Figure 9b-d). Thus, we confirmed our previous conclusion that vorinostat has a more moderate effect on protein mobility than does TSA.

### Kinetic modelling of HP1β, RPA194, and UBF dynamics with modulated transcription levels

**Figure 10:**
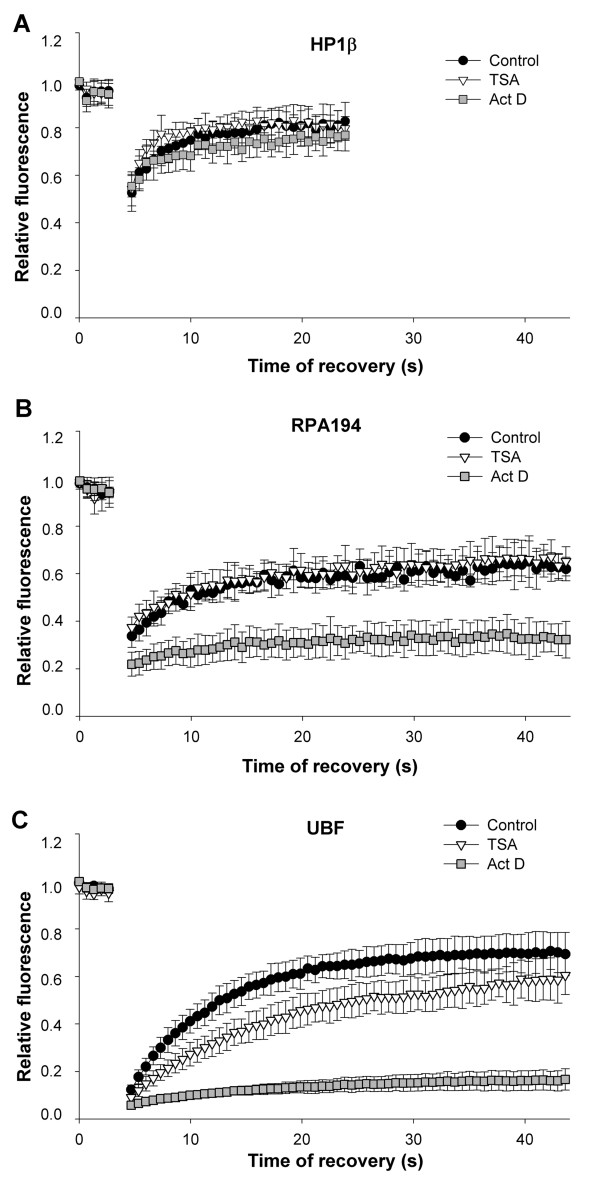
**Heterochromatin protein 1β (HP1β), RNA polymerase I large subunit (RPA194), and upstream binding factor (UBF) kinetics within nucleoli**. Fluorescence recovery time after photobleaching was studied for (a) HP1β, (b) RPA194, and (c) UBF proteins accumulated into nucleoli. Comparison was performed for given proteins studied in control (black dots), TSA-treated (triangles), and actinomycin D-stimulated (squares) MEFs.

Using the FRAP technique and subsequent statistical analysis, we compared the fluorescence recovery kinetics of HP1β, polymerase I subunit RPA194, and UBF in the nucleoli of untreated MEF cells and those treated with TSA and actinomycin D (Figure 10). Within nucleoli, HP1β ([*R*_10_] = 74%) recovered more rapidly than RPA194 ([*R*_10_] = 53%) and UBF ([*R*_10_] = 41%) (comparison of controls in Figure 10a-c). HP1β recovery time after photobleaching in nucleoli was not significantly changed by hyperacetylation or the reduced transcription levels caused by actinomycin D (Figure 10a). However, the recovery fluorescence for RPA194 was reduced after actinomycin D treatment, but not after TSA cell stimulation (Figure 10b). In the case of UBF, actinomycin D treatment completely reduced UBF diffusion (Figure 10c) and TSA also significantly decreased the diffusion of UBF protein (Figure 10c). Overall, our results show that actinomycin D significantly decreased protein mobility after photobleaching, while the effects of TSA were protein-specific and nuclear pattern-specific.

## Discussion

In the present work, we have uncovered a link between the dynamics and trajectories of chromatin-related proteins, and transcriptional regulation by TSA or an inhibitor of transcription elongation, actinomycin D. We have also addressed the effect of vorinostat (SAHA), another HDAC inhibitor that has promising therapeutic applications in oncology.

Cheutin *et al. *[30] reported distinct kinetics for HP1 subtypes (HP1α, HP1β, HP1γ) depending on whether they are accumulated in clusters of heterochromatin or are in euchromatin. Meshorer *et al. *[31] published that the recovery of heterochromatin-related HP1α is more rapid in mESCs than in terminally differentiated neurons derived from mESCs. Here, we report that in nucleoli, where HP1β appears in dispersed form [35], the kinetics of HP1β are similar to those described by Cheutin *et al. *[30] at euchromatin (Figure 6d,e). According to these results, it is evident that protein density and protein affiliation with heterochromatin or euchromatin influence protein mobility. This is illustrated by the observations that proteins recovered more slowly when accumulated in foci (HP1β, BMI1, TRF1) than when evenly dispersed throughout the nucleoplasm (Figures 6, 7, 8, 9). Moreover, the values for fluorescence recovery correlated with protein molecular weight only for proteins that were homogeneously distributed throughout the nucleoplasm (Figure 2b). These data suggest that it is not molecular weight alone, but molecular weight together with nuclear pattern that influences protein kinetics. This is supported by the lack of correlation between molecular weight and recovery of 18 proteins that were analysed irrespective of nuclear pattern (Figure 2a). Our results are consistent with Jacobson and Wojcieszyn [36], who reported that diffusion coefficients do not depend on molecular weight. However, Braga *et al. *[37] published that diffusion coefficients are lower in the nucleoplasm for higher molecular weight dextran, which is a temperature-dependent effect. Based on these results, it seems that biologically inactive molecules move differently than physiologically active proteins of similar size [38-40].

We also showed that, for all examples tested, suppression of transcription by actinomycin D had a strong impact on HP1β, BMI1, and TRF1 mobility. It is evident that actinomycin D, an agent that intercalates into double stranded DNA, robustly disturbed nuclear structure and induced changes in the mobility of proteins. Intriguingly, we observed more pronounced differences in the recovery time of HP1β and BMI1 in heterochromatic foci than in euchromatin regions after treatment with actinomycin D or HDAC inhibitor (Figures 6 and 7). For the most part, distinct global transcription levels were associated with decreased recovery kinetics of the studied proteins. Generally, protein residence time was more frequently reduced when core histones were hyperacetylated ([26]; Figures 7b,e and 8e). Similarly to Cheutin *et al. *[30], we observed that HP1β was an exception to this rule. In this case, histone hyperacetylation-induced by TSA significantly increased the mobility of HP1β in heterochromatic foci (Figure 6f). This supports previous data on the effect of HDAC inhibitors (TSA and sodium butyrate) on heterochromatin; HDAC inhibitors disturb the nuclear arrangement of HP1 subtypes and HP1 colocalisation within centromeric heterochromatin [41,42]. In contrast, we show here that vorinostat had a more moderate effect on protein mobility than TSA. Thus, the analysis of protein mobility after cytostatic treatments might reveal important side effects of clinically used drugs.

Interestingly, when we increased the transcriptional activity of ribosomal genes along with increased histone acetylation by treating cells with TSA [35], we observed no effect on RPA194 recovery time, similar to what was observed for HP1β in nucleoli (Figure 10a,b). However, a significantly decreased recovery time was observed for UBF after TSA treatment (Figure 10c). Because RPA194, UBF, and HP1β proteins are all associated with active transcription of ribosomal genes [11,35], these distinctions may be consequences of protein quantity at particular genomic regions and/or related to protein binding efficiency to other proteins or DNA in this region. Similarly, the local concentrations of proteins can influence the mobilisation and kinetics of particular proteins, as discussed in two papers by Ayoub *et al. *[43,44], especially for HP1β recruited to UV-damaged chromatin. Another explanation is that the recovery kinetics after photobleaching may be influenced by cell cycle changes. For RNA polymerase I, the recovery kinetics decrease as transcription increases during the cell cycle [19]. In S-phase, there is a reduced recovery after photobleaching of RPA194 compared with G1 cells [19]. Similarly, the low recovery properties of histone H2B and H3 ([31], and Figure 2a,b) were significantly reduced when proliferating mESCs were induced to neuronal differentiation, characterised by a cell cycle block in G0 phase [31]. As stated by Melcer and Meshorer [45], this phenomenon is not without exception. For example, the recovery kinetics of histone H3.3 is similarly hypodynamic in both pluripotent mESCs and after neuronal differentiation. Moreover, no differences in histone H1^o^ kinetics were observed during different cell cycle stages [31].

Our experiments were directed towards the detailed detection of trajectories of selected protein foci. We also measured a correlation between TRF1 foci trajectory and transcriptional activity, but it was not upheld for HP1β and BMI1 (Figures 3, 4, 5). This is similar to data published for nuclear gene positioning with respect to gene expression. For example Meaburn and Misteli [46] identified several genes that are spatially repositioned during breast cancer tumorigenesis, but observed gene activity-independent genome repositioning in the early stages of tumour formation. Recently, we showed that the nuclear radial position of the pluripotency gene Oct3/4 did not change when downregulated [47]. Thus, it seems that nuclear radial position is highly gene specific and likely depends on the transcriptional activity of surrounding chromatin and/or, as stated by Küpper *et al. *[48], radial chromatin positioning is shaped by local gene density, not by gene expression.

Together, the trajectories of HP1β, BMI1, and TRF1 foci were, in many cases, influenced by histone hyperacetylation and by suppression of transcription by actinomycin D (Figures 3, 4, 5). However, the localised dynamics or extended dynamics of other nuclear domains, such as PML bodies, are not changed after inhibition of RNA polymerase II by α-amanitin [49]. Here, we found that a protein trajectory was dependent on foci positioning; it matters whether the foci were located at the nuclear periphery or in the nuclear interior (Figures 3 and 4). Heterogeneity in the mobility of protein foci has also been reported by Guan *et al. *[50] for α1B-adrenoceptor in living cells. Moreover, increased energy dependent motion was observed by Wang *et al. *[28] for shorter telomeres when compared with longer and uncapped telomeres. Here, we show that both the trajectory and the average velocity were specific to nuclear positioning for HP1β, BMI1, and TRF1 foci (Table 1). This heterogeneity in foci movement is consistent with the observation of 'calm' and 'jittering' telomeres in live cells [28]. Similarly, Muratani *et al. *[49] have published velocity differences between individual PML bodies. Trajectories of PML bodies were defined as quasilinear, which is distinct from the trajectories of HP1β, BMI1, and TRF1 foci (Figures 3, 4, 5). These data demonstrate protein-specific kinetics and trajectories that likely reflect several biological events.

## Conclusions

We have described protein dynamics that can be influenced by several factors, including binding and release constants, residence times, diffusion coefficients [40], chromatin condensation [30], cell differentiation [45], and cell cycle-dependent transcriptional level [19]. In addition, Odenheimer *et al. *[51], suggest there are strong effect of chromatin nanostructure on local mobility. In our experimental model, no single factor was directly responsible for slow or fast fluorescence recovery after photobleaching. Thus, we propose that protein dynamics are likely influenced by several factors and cellular processes that combine to determine the kinetic properties of chromatin-related proteins.

## Methods

### Cell culture

Immortalised wild-type (wt) MEFs originated in the Laboratory of Professor Thomas Jenuwein, Max-Planck Institute of Immunobiology and Epigenetics, Freiburg, Germany. HeLa cells expressing histone H2B-GFP were a generous gift from Dr Marion Cremer (Ludwig-Maximilians University, Munich, Germany) and photoconvertible Dendra2 was used to label histone H4 in HepG2 cells (obtained from Professor Ivan Raška, Institute of Cellular Biology and Pathology, First Faculty of Medicine, Charles University, Prague, Czech Republic). U2OS cells expressing BMI1-GFP were a generous gift from Associate Professor Dušan Cmarko (Institute of Cellular Biology and Pathology, First Faculty of Medicine, Charles University, Prague, Czech Republic). Dr Paul Verbruggen (Swammerdam Institute for Life Sciences, University of Amsterdam, Amsterdam, The Netherlands) provided 3T3 cells with stable HP1β expression. The majority of cells were cultivated in high-glucose Dulbecco's modified Eagle medium (DMEM) containing 10% sera, but MEFs were cultivated according to Harničarová Horáková *et al. *[35] and Dendra-HepG2 were grown in low-glucose DMEM supplemented with 10% sera. When the cultures reached 70% confluence, the cells were treated with a final concentration of 100 nM trichostatin A (TSA; Sigma-Aldrich, St Louis, MO, USA) and 0.5 μg/ml actinomycin D (#A9415, Sigma-Aldrich). The hyperacetylation effects of TSA have been verified elsewhere [41,52] and actinomycin D is a commonly used reagent to block transcription elongation mediated by RNA polymerases [20,21,53].

The mESC line, GOWT1 (a generous gift from Hitoshi Niwa, Laboratory for Pluripotent Stem Cell Studies, RIKEN Center for Developmental Biology, Kobe, Japan), was cultivated in standard mESC medium (Glasgow minimum essential medium (GMEM) + 10% foetal calf serum (FCS)) with leukaemia inhibitory factor (LIF). These cells were maintained in the presence of puromycin (1.5 μg/ml) to select for Oct3/4 positive undifferentiated stem cells. All cell cultures were maintained at 37°C in a humidified atmosphere containing 5% CO_2_.

### Plasmids

The plasmids were transformed into *Escherichia coli *DH5α for amplification. Plasmid DNA was isolated using the QIAGEN Large-Construct kit (#12462; QIAGEN, Bio-Consult, Prague, Czech Republic). For transfection of MEFs we used the METAFECTENE PRO system (Biontex Laboratories GmbH, Planegg, Germany). The following plasmids were used in this study: JMJD2b-GFP (obtained from Professor Thomas Jenuwein and Dr Nicholas Shukeir, Max-Planck Institute of Immunobiology, Freiburg, Germany); HP1α-GFP and HP1β-GFP (from the laboratory of Dr Tom Misteli, National Institutes of Health, Bethesda, MD, USA); RPA194-GFP (Addgene Inc., Cambridge, MA, USA; #17660); UBF-GFP (Addgene; #17656); Ubiquitin-GFP (Addgene; #11928); p53-GFP (Addgene; #12091); pmCherry-alpha-tubulin-IRES-puro2 (Addgene; #1360); lamin A-GFP (Addgene; #17662); c-myc-GFP (a gift from Dr Hiroyoshi Ariga, Hokkaido University, Graduate School of Pharmaceutical Sciences, Kita-Ku, Sapporo, Japan), β-catenin-enhanced GFP and STAT1-GFP (gifts from Dr Vítězslav Bryja and Dr Jiří Pacherník, Faculty of Sciences, Masaryk University, Brno, Czech Republic). GFP-hTRF1-pS65T-C1 was a gift from Dr C M Counter, Duke University Medical Center, Durham, NC, USA. GFP-PML was obtained from Douglas Durso, Customer Service Representative, Roche NimbleGen, Inc., Madison, WI, USA. pEGFP-C1 cloning plasmid was purchased from Clontech Laboratories, Inc. (Mountain View, CA, USA).

### Single particle tracking analysis

Time series of images were acquired on a confocal Leica TSC SP 5× microscope (Leica, Mannheim, Germany). We captured images every 2.6 s for 20 min. The image analysis was performed using Matlab software http://www.mathworks.com/products/matlab/. Each frame was cropped to contain a single cell. Weighted centres of foci were computed for every frame (see below). The centres formed point sets, which were used to compensate for cellular movement, as described in [54]. It was impossible to localise all foci in all frames (mostly due to out-of-focus motion). Therefore, the trajectories were made in two steps: (1) all corresponding points in successive frames were linked together. The correspondence was identified by a point-based alignment algorithm. This step produced many foci subtracks. In this step, we determined the area of the smallest ellipse required to enclose all points in each subtrack. (2) We combined subtracks in one track if their minimal ellipses intersected and followed each other in time. The collection of subtracks defined the trajectory of a focus. We calculated the average velocity of the foci as the length of all trajectory subtracks divided by the sum of the subtracks' time spans. We measured the area of the minimal enclosing ellipse of all trajectory points. We studied the foci trajectories in the interior of nucleus, as well as at the nuclear periphery. The trajectories that had a local radius less of than 60% for the initial frame were taken to be the central trajectory. Local radius has been defined elsewhere [55].

We calculated the weighted centres of the foci using the following procedure. First, to remove noise, each frame of a sequence was filtered through a median filter with a circular window of 7 pixels. The image was then smoothed by convolution with Gaussian kernel (σ = 3). Next, we computed the morphological *h*-dome transform with manually selected *h *value. Only domes containing a pixel intensity exactly equal to *h *were considered objects of interest. This was achieved using morphological reconstructions of *h*-dome images from seeds corresponding to pixels of intensity *h*. Finally, we computed the weighted centres within *h*-domes of detected objects (foci).

To compute the local radius of points, we had to segment the nucleus, which was performed using the following algorithm. First, the image was smoothed by convolution with a Gaussian kernel σ = 10), and then the threshold was adjusted using the chord method [56]. Morphological closing of a suitable radius was performed to fill the holes.

We calculated MSD and diffusion coefficient as previously described [57]. We used a modified MSD formula for sequences with missing probes as previously described [57]. The MSD curve was calculated for all trajectories up to the time lag of 100 frames (260 s). We present average curves for different cells in Figure 5.

The diffusion coefficient was estimated by linear fitting to the MSD curve. The optimal number of fitting points was determined using the iterative scheme proposed in [57]. We calculated the diffusion coefficient from the average MSD curves.

### Live cell imaging, FRAP analysis, and statistics

Cells growing on 50-mm glass-bottomed dishes (MatTek, Corporation, Ashland, MA, USA; #P50G-0-30-F) were placed in a cultivation hood (EMBL, Heidelberg, Germany) with a stable temperature of 37°C and 5% CO_2_. For live cell studies, we used a confocal Leica TSC SP-5× microscope, equipped with a white light laser (470 to 670 nm); argon laser (488 nm), and UV lasers (355 nm and 405 nm); 64× magnification and numerical aperture NA = 1.4 were used. The movement of individual cells and chromatin-related proteins, either accumulated in distinct foci or dispersed throughout the nucleoplasm, was monitored using Leica LAS AF software (version 2.1.2). For trajectory analysis, the cells were monitored for 20 min; scanning was performed every 2 s. For FRAP, GFP was excited at 488-nm using an argon laser. During FRAP, fluorescent molecules were bleached at defined regions of interest (ROI) (2 μm^2^). Bleaching was performed with a 488-nm argon laser for 1.2 s. For scanning, we used 10% laser intensity and for FRAP we used 100% laser power. To minimise the laser instability that can be caused by external temperature, the room temperature was stabilised using a highly precise air conditioner (room temperature was 21°C to 22°C). Moreover, a particular set of experiments was analysed by FRAP in a single day to minimise the effects of laser instability. Prebleaching was set to five frames (each frame is 0.66 s), and the rate of fluorescence recovery provided information about the rate at which the fluorescent molecules moved into the bleached region. The cells were monitored over 0.66 s intervals, for 25 s.

To study correlations, we used linear regression analysis as a statistical tool in the SigmaPlot software (version 8.0; SPSS, Chicago, IL, USA). Potential correlation between molecular weight (MW) and fluorescence recovery after photobleaching was evaluated by the Pearson's product-moment correlation coefficient (r). We constructed linear regression curves and a confidence interval around (r) that showed 95% probability of statistically significant results.

## Competing interests

The authors declare that they have no competing interests.

## Authors' contributions

LS was responsible for cell cultivation, transfection, FRAP experiments, statistics, and regression analysis, and writing the manuscript. EB conceived experiments and wrote the manuscript. PM and OD analysed trajectories of selected proteins and calculated diffusion coefficients and MSD; they are responsible for the results in Figures 3, 4, 5 and Tables 1, 2, 3. The algorithm for trajectories was provided by OD. SL is responsible for the western blot data. SK interpreted data and provided financial support.
